# Cytolytic and systemic toxic effects induced by the aqueous extract of the fire coral *Millepora alcicornis* collected in the Mexican Caribbean and detection of two types of cytolisins

**DOI:** 10.1186/s40409-015-0035-6

**Published:** 2015-09-25

**Authors:** Rosalina Hernández-Matehuala, Alejandra Rojas-Molina, Alma Angelica Vuelvas-Solórzano, Alejandro Garcia-Arredondo, Cesar Ibarra Alvarado, Norma Olguín-López, Manuel Aguilar

**Affiliations:** Posgrado en Ciencias del Mar y Limnología, Universidad Nacional Autónoma de México, Mexico City, Mexico; Laboratorio de Investigación Química y Farmacológica de Productos Naturales, Facultad de Química, Universidad Autónoma de Querétaro, Centro Universitario, Querétaro, 76010 Mexico; Instituto de Neurobiología, Universidad Nacional Autónoma de México, Campus Juriquilla, Querétaro, 76201 Mexico

**Keywords:** Fire coral, *Millepora alcicornis*, Hemolysis, Systemic toxicity, Electronic microscopy

## Abstract

**Background:**

*Millepora alcicornis* is a branching hydrocoral common throughout the Caribbean Sea. Like other members of this genus, this species is capable of inducing skin eruptions and blisters with severe pain after contact. In the present study, we investigated the toxicity of the *M. alcicornis* aqueous extract on several animal models. Considering that some cnidarian hemolysins have been associated to local tissue damage, since they also induce lysis of other cell types, we also made a partial characterization of the hemolytic activity of *M. alcicornis* aqueous extract. This information is important for understanding the defense mechanisms of the “fire corals”.

**Methods:**

The effects of pH, temperature, and some divalent cations on the hemolytic activity of the extract were assayed, followed by a zymogram analysis to detect the cytolysins and determine their approximate molecular weight. The toxicity of the aqueous extract was assayed in mice, by intravenous administration, and histopathological changes on several tissues were analyzed by light microscopy. The toxicity of the extract was also tested in *Artemia salina* nauplii, and the damages caused on the crustaceans were analyzed by transmission and scanning electron microscopy.

**Results:**

The hemolytic activity of the hydrocoral extract was enhanced in the presence of Ca^2+^ (≥2 mM), Mg^2+^ (≥6 mM), and Ba^2+^ (≥0.1 mM); however, it was reduced in the presence of Cu^2+^ (≥0.1 mM), Zn^2+^ (≥6 mM), and EDTA (≥0.34 mM). Differences in the pH did not affect the hemolytic activity, but it was temperature-sensitive, since preincubation at ≥ 50 °C sharply reduced hemolysis. The zymogram showed the presence of two types of hemolysins: ~ 28–30 kDa proteins with phospholipase A_2_ activity and ~ 200 kDa proteins that do not elicit enzymatic activity. The aqueous extract of this cnidarian was lethal to mice (LD_50_ = 17 μg protein/g), and induced kidney, liver, and lung damages. Under denaturing conditions, the aqueous extract completely lost its toxic and hemolytic activities.

**Conclusions:**

The results showed that the *M. alcicornis* aqueous extract contains two types of thermolabile hemolysins: proteins of approximately 28–30 kDa with PLA_2_ activity, while the others are larger proteins of approximately 200 kDa, which do not possess PLA_2_ activity. Those thermolabile cytolysins, which are stable to pH changes and whose activity is calcium dependent, are capable of inducing damage in lung, kidney and liver tissues, resulting in a slow death of mice. *M. alcicornis* cytolysins also provoke tissue dissociation in *Artemia salina* nauplii that might be attributed to pore forming mechanisms.

## Background

Mexican marine biodiversity is extremely rich and the coral reefs of the Mexican Caribbean own a particularly high number of species; these include hydrozoans with calcareous exoskeleton of the genus *Millepora* (phylum Cnidaria; class Hydrozoa), which are the second-most abundant reef-forming organisms in coral reef ecosystems [1]. Like all cnidarians, *Millepora* species are armed with stinging cells, cnidocytes, that hold nematocysts, which consist of a capsule filled with venom that is injected through a tubule when a physical or chemical stimulus is detected [2–4].

*Millepora* spp. are commonly known as “fire corals” since they can puncture the human skin and produce lesions similar to burns. These injuries provoke irritation, burning or stinging pain, erythematous and edematous dermatitis, pruritus, hives, and skin necrosis. Systemic symptoms consist of malaise, nausea, vomiting, abdominal pain, muscle spasm, diarrhea, respiratory difficulty, tachycardia, hypotension, and fever [1, 5]. A clinical case of severe systemic toxicity due to *Millepora* spp. envenomation has been reported, including nephrotic syndrome, acute renal failure, and pulmonary edema [6].

Despite their ecological and toxicological importance, few studies have been conducted on *Millepora* species. Early investigations of *M. tenera* demonstrated that the crude extracts of these fire corals exhibited hemolytic and dermonecrotic properties, and are highly toxic in mice when intravenously administered, inducing dyspnea, convulsions, and death [7, 8]. Later, it was found that *M. plathyphylla* and *M. dichotoma* venoms were lethal to mice and had hemolytic, dermonecrotic, vasopermeable, and antigenic properties [9, 10]. In another study, Iguchi et al. [11] isolated a 18-kDa cytotoxin from nematocysts of *M. dichotoma* var. *tenera*. This cytotoxin, named MCTx-1, was toxic to L1210 mouse leukemia cells and was lethal to cryfish. The primary structure of MCTx-1 was deduced from the corresponding cDNA [11].

Studies carried out by our research group showed that the venom of *M. complanata* provokes hemolysis, displays PLA_2_ activity, and contains proteins that induce calcium-dependent contractions in guinea pig ileum and in rat aorta [12, 13]. Hemolytic and PLA_2_ activities were abolished when *M. complanata* crude venom extract was incubated in a boiling water bath for 20 min. However, although contractile effects on intestinal and arterial smooth muscle were reduced, they were not completely blocked [14].

Concerning *M. alcicornis*, an extract obtained by soaking specimens from Florida in Sörensen buffer caused extensive hemolysis and death in mice after several hours [7]. Chromatography on DEAE-cellulose and Sephadex G-100 of the extract led to the partial purification of a protein (approximately 100 kDa) with a 14-fold increased toxicity [15]. Recently, our group demonstrated that the aqueous extract of *M. alcicornis* induced hemolysis in rat erythrocytes, exhibited PLA_2_ activity and elicited a concentration-dependent contraction of isolated rat aortic rings [14]. Although the toxic and lethal effects of *M. alcicornis* have been previously reported, to date the identity of the toxins, which cause the systemic toxicity and the key affected tissues are unknown.

In the present study, we assessed the lethality, systemic toxicity and histopathological effects induced in mice by the aqueous extract of *M. alcicornis* collected in the Mexican Caribbean. We also evaluated the toxicity of this extract to brine shrimp *Artemia salina* nauplii. The damages caused by the extract on crustacean and erythrocyte membranes were analyzed by scanning electron microscopy (SEM) and transmission electron microscopy (TEM). In addition, the molecular weights of the toxins responsible for the hemolytic and PLA_2_ activities are reported for the first time.

## Methods

### Materials

Citric acid, sodium citrate, and *p*-bromophenacyl bromide (*p*-BPB) were obtained from Sigma, USA. Salts and other reagents were obtained from J.T. Baker, USA. The reagents used in the determination of protein concentration and electrophoresis were from Bio-Rad, USA.

### Sample collection and aqueous extract preparation

Fragments of *M. alcicornis* were collected by scuba diving from coral reefs in the area known as “La Bocana Chica” that belongs to the Parque Nacional Arrecifes de Puerto Morelos (Quintana Roo, Mexico) in November 2008 at depths of 4 to 10 m. The fragments were immediately frozen in dry ice and transported to our laboratory where extraction was carried out.

Nematocyst discharge was accomplished by stirring the hydrozoan fragments in deionized water (pH 7) at 4 °C for 18 h. The extract obtained was centrifuged at 2060 *g* for 15 min at 4 °C. This procedure was repeated twice, and the supernatant was lyophilized and stored at −70 °C. The lyophilized supernatant was dissolved in deionized water at 150 mg/mL, and was centrifuged at 2060 *g* for 15 min at 4 °C. The supernatant was filtered through a 0.45 μm pore filter (Millipore). The filtrate was stored at −20 °C until its use in the bioassays. Protein content was determined according to the method of Bradford, using a standard curve prepared with lyophilized bovine serum albumin [16].

### Hemolytic activity

The hemolytic activity of the *M. alcicornis* aqueous extract was monitored according to a method previously described with some modifications [17]. Briefly, samples for the assay contained a mixture (1 mL) of Alsever’s solution (120 mM D-glucose, 30 mM sodium citrate, 7 mM NaCl, and 2 mM citric acid; pH 7.4) with 50 μL of a 1 % suspension of erythrocytes (from rat, rabbit, human, chicken, mouse, or guinea pig) and the required volume of the extract. These samples were incubated at 37 °C for 30 min. After centrifugation at 1430 *g* for 4 min at 4 °C, the A_415_ nm of the supernatant fluid containing the hemoglobin released from lysed erythrocytes was measured in a spectrophotometer (Lambda Bio, Perkin Elmer, USA). Each experiment was normalized with respect to complete hemolysis, which was measured by diluting the erythrocyte sample in deionized water instead of Alsever’s buffer. One hemolytic unit (HU_50_) was defined as the amount of protein sample required to cause 50 % hemolysis.

### Effect of temperature on the hemolytic activity

In order to analyze the effect of temperature on the hemolytic activity of the aqueous extract, the hemolysis assay was performed at various incubation temperatures (0 to 100 °C) for 30 min. For thermal stability analysis, samples of the aqueous extract were preincubated at different temperatures (0 to 100 °C) for 60 min.

### Effect of pH on the hemolytic activity

Samples of the lyophilized extract were incubated for 24 h at 4 °C with several buffers at a pH range from 0 to 13, namely 0.2 M KCl-HCl, 0.02 M acetate–acetic acid, 0.02 M Tris–HCl, 0.02 M sodium borate–boric acid, and 0.025 M NaHCO_3_-NaOH. After incubation, the hemolytic effect of the extract was assayed (*n* = 3).

### Effect of different divalent cations on the hemolytic activity

Different divalent cations (Ca^2+^, Mg^2+^, Ba^2+^, Cu^2+^, and Zn^2+^) and ethylenediaminetetraacetic acid (EDTA) were added to the Alsever’s solution and the hemolytic effect of the extract was assayed (*n* = 3).

### Effect of a PLA_2_ inhibitor on the hemolytic activity

Inhibition of hemolysis was evaluated after incubating the extract with 0.3, 1 or 3.3 mM *p*-BPB in the Alsever solution for 22 h at 4 °C.

### Electrophoresis and zimography

Sodium dodecyl sulfate polyacrylamide gel electrophoresis (SDS-PAGE) was carried out as previously described [18]. Samples of 10 μg protein were run on 12 % polyacrylamide gels at 90 V for 30 min and then at 120 V for 2 h at 4 °C, with tris-glycine as buffer under reducing and non-reducing conditions. Protein bands were visualized with Coomassie blue. Molecular masses were determined against broad-range and low-range polypeptide standards (Bio-Rad, USA).

Proteins with PLA_2_ activity were identified and their molecular weights were determined using a zymography technique with some modifications [19]: the aqueous extract (50 and 100 μg protein/mL) was run on a 12 % polyacrylamide gel at 90 V at 4 °C under non-reducing conditions. Then, the gel was washed with 100 mM Tris–HCl, pH 7.4 (with 1.0 % Triton X-100), during 1 h to remove traces of sodium dodecyl sulfate and it was incubated for 15 h at room temperature with 50 mM Tris–HCl, pH 7.4 (with 140 mM NaCl and 2.5 mM CaCl_2_), over a 2 % agarose gel prepared with 50 mM Tris–HCl and 6 % egg yolk. PLA_2_ from honey bee venom (*Apis mellifera*) was used as a positive control.

The proteins which displayed hemolytic activity were detected and their molecular weights were determined using the modified zymography technique. The gel was electrophoresed with the extract at 90 V at 4 °C under non-reducing conditions and after that it was washed with 100 mM Tris–HCl for 1 h. Thereafter, the gel was incubated for 4 h at room temperature over a 1.5 % agar gel; which was prepared by adding egg yolk (5 %) at 60 °C, and washed erythrocytes (3 %) added at 40 °C, on phosphate buffered saline (PBS) (pH 7.4) supplemented with CaCl_2_ 10 mM. The presence of a clear band in the agar gel indicated the presence of hemolysins.

### Caseinolytic activity

Caseinolytic activity was assayed according to the method proposed by Murata et al. [20]. Briefly, 0.4 mL of casein (2 % in 0.2 M Tris–HCl buffer pH 8.5) was incubated in the presence of the extract at 20, 40, 60, 80, and 100 μg protein at 37 °C for 2 h. The reaction was stopped by adding 1.5 mL of 0.44 M trichloroacetic acid. The mixture was allowed to stand for 30 min, and then centrifuged at 1500 *g* for 15 min. An aliquot (1.0 mL) was mixed with 2.5 mL of 0.4 M sodium carbonate and 0.5 mL of 1:2 diluted Folin’s reagent and the color developed was read at 660 nm. One unit of the enzymatic activity was defined as the amount of enzyme required to increase the absorbance by 0.01 at 600 nm/h at 37 °C. Activity was expressed as units/h at 37 °C.

### Lethality assay in mice

For this assay, CD1 male mice (weighing 20 g ± 3) were supplied by the Animal House of the Institute of Neurobiology (Universidad Nacional Autónoma de México). The animals were maintained with free access to standard mouse food pellets and water *ad libitum*. The *M. alcicornis* aqueous extract was administered by tail vein injection (*n* = 3) at different doses (0.16, 0.32, 1.5, 3.0, 6.0, 12, 24, and 48 μg protein/g of body weight) dissolved in 200 μL of saline solution. Additionally, the protein contained in the aqueous extract was denatured by incubation in a boiling water bath for 20 min and injected employing the same concentration range. Deaths occurring within 24 h were recorded and the mean lethal dose (LD_50_) was estimated by a Probit analysis. Control mice were treated with 200 μL of saline solution. All experiments were performed in accordance with the Mexican Official Standard NOM-062-ZOO-1999 for the production, care and use of laboratory animals.

### Histopathological analyses

Mice that survived the intravenous administration of the intact and denatured (extract incubated in a boiling water bath for 20 min) aqueous extracts of *M. alcicornis*, including the control group, were sacrificed by cervical dislocation at the end of the experimental period (24 h after the extract administration). Lung, kidney, liver, heart, skeletal muscle and brain samples were dissected out and fixed in 4 % formaldehyde and dehydrated in an increasing alcohol series, cleared in xylene series, and embedded in paraffin blocks. These blocks were cut into 5–6 μm thick sections, using a microtome Ecoshel 335, which were prepared and stained with hematoxylin-eosin for routine histological examination by light microscopy (LM) with a Leica DM500 microscope.

### Lethality test in *Artemia salina*

*M. alcicornis* extract toxicity was evaluated for lethality in brine shrimp larvae according to a procedure previously described [21]. Briefly, 25 mg of dried brine shrimp eggs were placed in saline medium (Instant Ocean, USA). After 48 h, the hatched shrimps were ready for testing. One-day-old larvae (ten per vial) were transferred into 5-mL vials containing the aqueous extract in saline solution. The extract was tested by triplicate at 0.01, 0.1, 1, 10, 100, and 316 μg protein/mL. After 24 h, the number of dead shrimps was counted and the percentage of mortality was calculated. From these data, the LD_50_ (μg of protein/mL) was calculated by a Probit analysis.

### Analysis by electron microscopy techniques

Samples of *A. salina* nauplii—employed in the lethality test—and samples of rat erythrocytes—obtained from the hemolysis assay—were examined for morphological alterations induced by the aqueous extract of *M. alcicornis*. Some samples were dyed with starch blue and observed directly through light microscopy. For observations by SEM and TEM, samples were fixed in filtered sea water or isotonic saline solution containing 3 % glutaraldehyde and 0.1 M of sodium cacodylate, postfixed in 2 % OsO_4_ in cacodylate buffer, and dehydrated in an ethanol series. Then, for TEM analysis, samples were embedded in Epon epoxy resin and the blocks obtained were sliced (60 nm) in an ultramicrotome (Mtx RMC Boeckler Instruments, USA) and contrasted with uranyl acetate and lead citrate. These sections were observed in an electron microscope (JEM 1010, JEOL, USA) operated at 80 kV. For SEM analysis, the samples were dried in a critical-point dryer (Polaron E5000, Quorum Technologies, UK), covered with carbon in an evaporator (JEE4X, JEOL, USA) and with a thin sheet of gold in an ion sputterer (Polaron 11-HD, Quorum Technologies, UK), and finally observed with a scanning electron microscope (DMS 950, Zeiss International) at an accelerating voltage of 20 to 25 kV.

### Data analysis and statistics

Results are expressed as the mean ± S.E.M. from three experiments. The LD_50_ was calculated by Probit analysis. For hemolytic activity, concentration-response curves (CRC) were repeated three times employing different animals including human samples. CRC were plotted and fitted to the Boltzmann equation using the data analysis and graphics program Prism (Graph Pad Software, USA). The values of the mean hemolytic concentrations (HU_50_) were obtained from the CRC. Statistical evaluation of the data was performed using one-way ANOVA, followed by a Tukey’s multiple comparison test. In all cases statistical significance is indicated by *p* < 0.05.

## Results

### Hemolytic activity

**Fig. 1:**
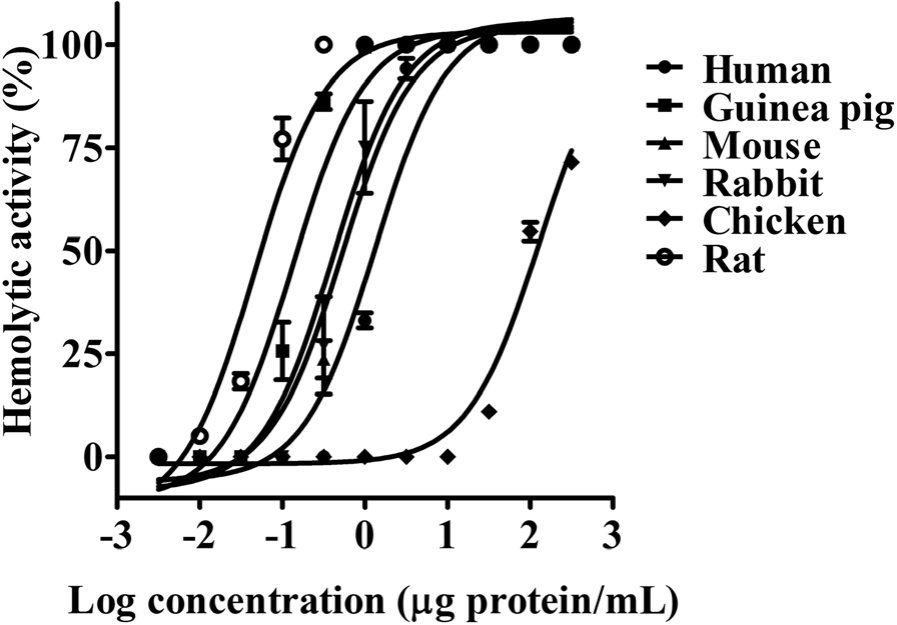
Concentration-response curves showing the hemolytic activity of the *M. alcicornis* aqueous extract on erythrocytes from various species

The *M. alcicornis* aqueous extract (0.0001 to 150 μg/mL) produced a concentration-dependent hemolysis on erythrocytes (Fig. 1) that was completely abolished after incubation in boiling water bath for 20 min. Chicken erythrocytes were markedly more resistant to the hemolytic effect of the *M. alcicornis* extract than the erythrocytes from other species (Table 1). In the present study rat erythrocytes were used to characterize the hemolytic activity of the extract. The hydrocoral extract (HU_50_ = 0.042 ± 0.005 μg protein/mL) was approximately 2200-fold more potent than ionomycin (HU_50_ = 92.57 ± 1.013 μg/mL), used as a control.

**Table 1:** Hemolytic activity of the *M. alcicornis* extract on several types of erythrocytes

Erythrocytes type	Hemolytic unit (HU_50_)
Rat	0.042 ± 0.005
Guinea pig	0.29 ± 0.15
Mouse	0.41 ± 0.015
Rabbit	0.63 ± 0.35
Human	1.0 ± 0.31
Chicken	129 ± 8.2*

*p < 0.05 versus hemolysis on rat erythrocytes

### Characterization of the hemolytic activity

**Fig. 2:**
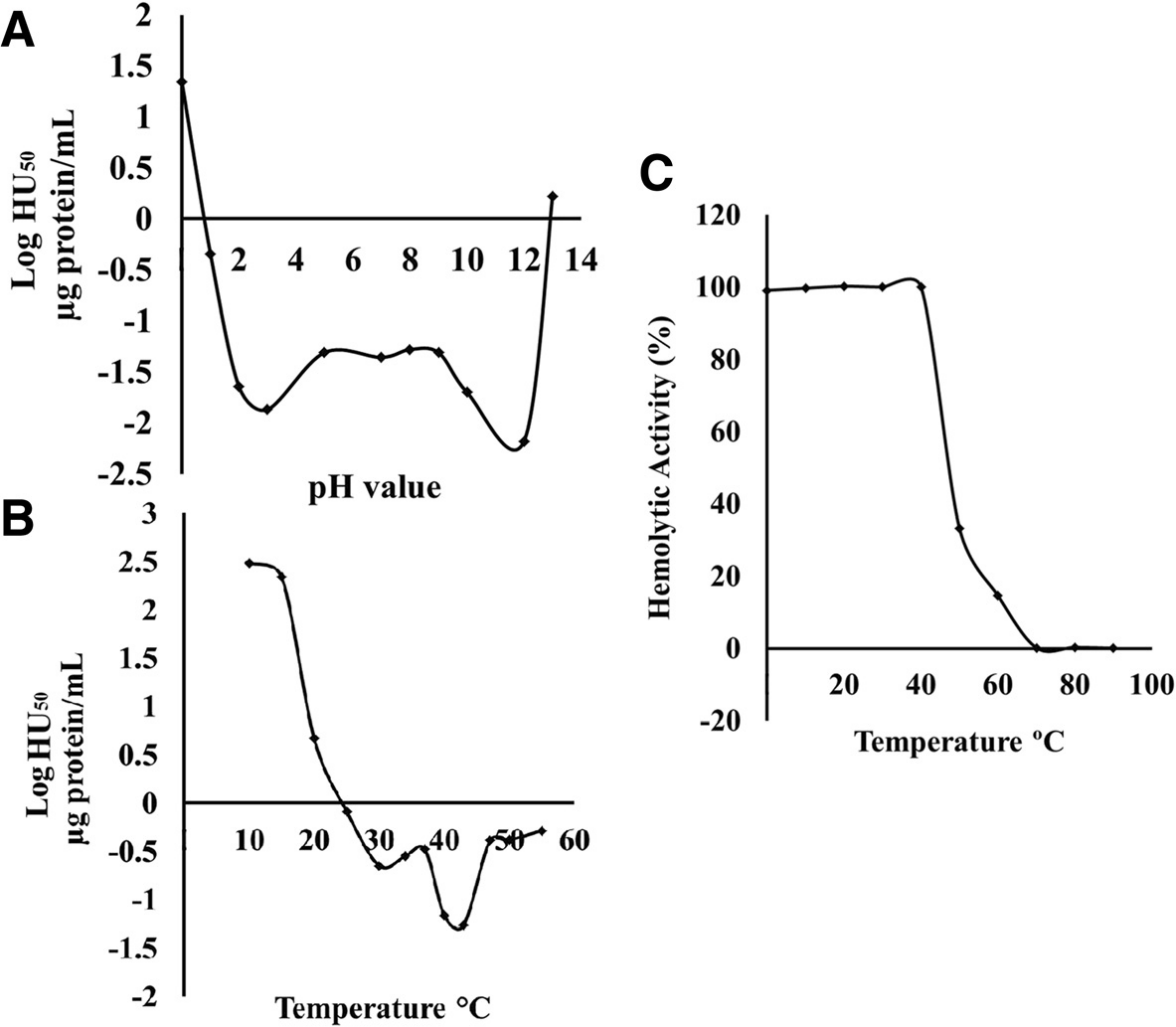
Effect of (**a**) pH and (**b**) incubation temperature on the hemolytic activity of the aqueous extract of *M. alcicornis*. **c** Stability of the hemolytic activity of the extract after its incubation at different temperatures

The hemolytic activity of the *M. alcicornis* aqueous extract was very stable with regard to pH (Fig. 2a). Concerning the effect of temperature, it was found that the highest hemolytic activity occurred at 40 to 43 °C, and temperatures lower than 20 °C provoked a decrease in potency and efficiency of the hemolysis (Fig. 2b). On the other hand, temperatures higher than 55 °C caused erythrocyte lysis (Fig. 2b and c). Analysis of the extract thermal stability showed that the hemolytic activity was inactivated at temperatures higher than 60 °C, while at preincubation temperatures lower than 45 °C, the hemolytic activity of the extract was conserved (Fig. 2c).

The hemolysis caused by the hydrocoral extract was assessed in the presence of divalent cations such as Ca^2+^, Mg^2+^, Cu^2+^, Zn^2+^, or Ba^2+^. These experiments showed that the hemolytic activity was depressed by Cu^2+^ 0.1 mM or Zn^2+^ 6 mM, however it was increased by addition of Ca^2+^ and Mg^2+^. In the case of Ba^2+^, concentrations lower than 6 mM increased the hemolytic activity of the extract, whereas concentrations higher than 8 mM induced inhibition of this activity (Table 2). Hemoysis produced by the extract was reduced after incubation with EDTA 0.34 mM and sharply disappeared at EDTA 0.43 mM (Table 3).

**Table 2:** Effects of divalent cations on the hemolytic activity of *M. alcicornis* aqueous extract tested at the HU_50_

Concentration (mM)	Hemolytic activity (%)				
	Ca^2+^	Mg^2+^	Cu^2+^	Zn^2+^	Ba^2+^
0.001	48 ± 9	54 ± 4	49 ± 4	66 ± 2	51 ± 6
0.01	49 ± 05	55 ± 1	40 ± 2	68 ± 2	54 ± 5
0.1	52 ± 1	48 ± 3	26 ± 3	65 ± 1	59 ± 6
1	51 ± 4	54 ± 4	29 ± 3	62 ± 0.0	79 ± 2
2	65 ± 4	61 ± 3	7 ± 4	46 ± 1	84 ± 4
4	76 ± 1	57 ± 6	0 ± 0	36 ± 1	81 ± 4
6	98 ± 2	63 ± 3		2 ± 6	83 ± 2
8	100 ± 0.0	73 ± 3		0 ± 0	1 ± 0.0
10	100 ± 0.0	87 ± 2		0 ± 0	0 ± 0.0
12		100 ± 0.00

**Table 3:** Effect of EDTA on the hemolytic activity of the *M. alcicornis* aqueous extract tested at the HU_50_

Concentration (mM)	Hemolytic activity (%)
4.3E-05	50 ± 2.0
0.0004	55 ± 7
0.0043	60 ± 3
0.043	58 ± 6
0.086	59 ± 5
0.172	59 ± 1
0.258	57 ± 3
0.344	45 ± 0.00
0.43	0.00 ± 0
0.5	0.00 ± 0

**Fig. 3:**
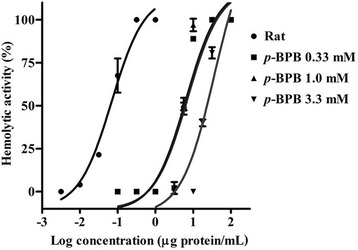
Concentration-response curves showing the effect of *p*-BPB, a PLA_2_ inhibitor, on the hemolytic activity of the *M. alcicornis* aqueous extract

The hemolytic activity of the *M. alcicornis* aqueous extract was significantly reduced after incubation with the PLA_2_ inhibitor *p*-BPB. In these experiments, the concentration response curves were rightward shifted depending on the concentration of the inhibitor. In the absence of *p*-BPB the HU_50_ was 0.069 ± 0.081 μg protein/mL, after incubation with 0.33 mM *p*-BPB the HU_50_ was 7.42 ± 0.13 μg protein/mL, with 1.0 mM *p*-BPB the HU_50_ was 6.51 ± 0.14 μg protein/mL. Finally, at 3.3 mM *p*-BPB a greater inhibitory effect was observed and the value of the HU_50_ increased up to 35.89 ± 0.16 μg protein/mL (Fig. 3).

### SDS-PAGE electrophoresis and zymography

**Fig. 4:**
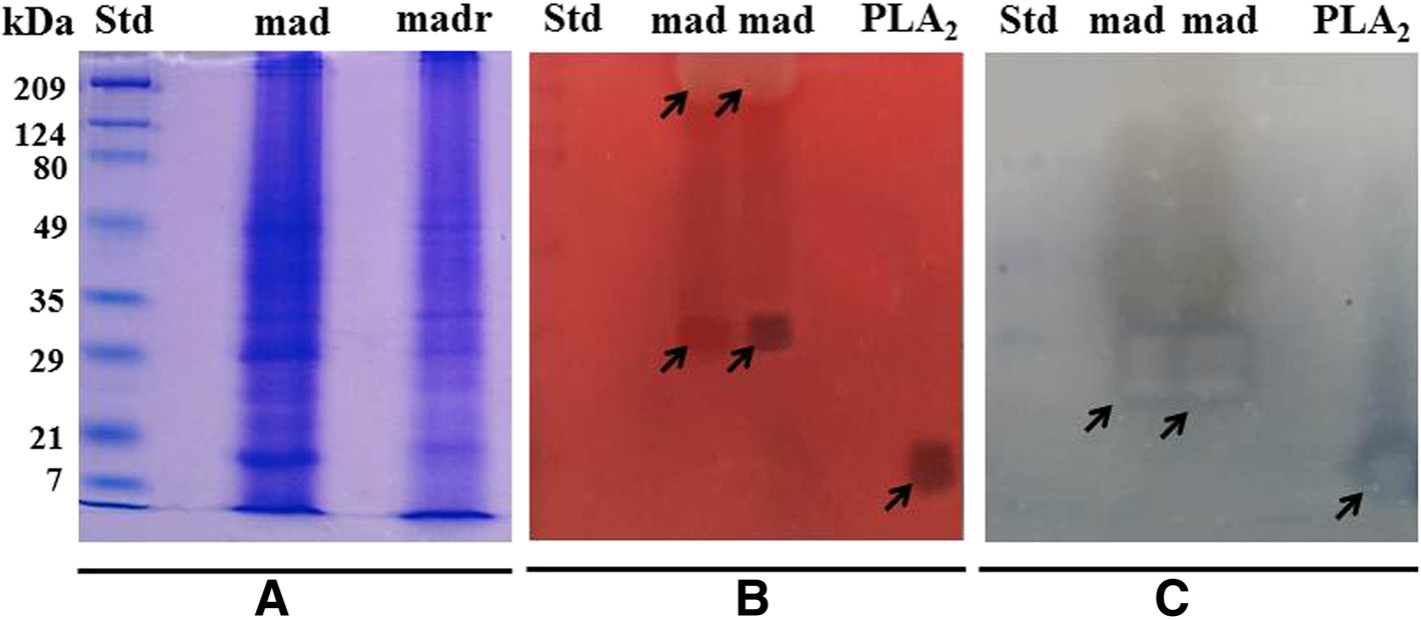
**a** Electrophoretic profile under non-reducing (Mad) and reducing (Madr) conditions. The protein bands were visualized by the Coomassie blue staining method. **b** Hemolysis zymogram on a 1.5 % agar gel supplemented with 5 % egg yolk and 3 % of washed erythrocytes, two hemolytic zones were observed in the gel corresponding to ~28-30 and ~200 kDa. **c** PLA_2_ zymogram on a 2 % agarose gel supplemented with 6 % egg yolk showed only one zone with enzymatic activity corresponding to ~28-30 kDa

The *M. alcicornis* aqueous extract contains proteins with a broad range of molecular weights, between 8 and 200 kDa and under reducing conditions, the electrophoretic profile was altered (Fig. 4a). Two main hemolytic zones were identified by zymography, one of which corresponded to a band of approximately 28–30 kDa, and the other one was related to a broad band of 200 kDa, approximately (Fig. 4b). Under reducing conditions, the 200 kDa band disappeared, which suggested that it consists of two or more dimers. The zymography analysis showed that the 28 kDa band possessed PLA_2_ activity, while the 200 kDa did not elicit this enzymatic activity (Fig. 4c).

### Lethality assay in mice and systemic effects

When intravenously injected, the aqueous extract of *M. alcicornis* was lethal to mice with a LD_50_ of 17 μg protein/g of body weight (Table 4). At doses lower than the LD_50_ (1.5 and 3.0 μg protein/g) the extract induced forced respiration immediately after the administration, but mice recovered within a few minutes. Mice also showed a progressive depressed responsiveness, which was usually pain related. However, they progressively recovered their normal behavior. At higher doses (6 and 12 μg protein/g), the aqueous extract also induced respiratory difficulty, hypoactivity, and other symptoms such as grooming, paralysis of the hind legs, intestinal inflammation, hemoglobinuria, and after a while, complete paralysis. In some cases, respiratory failure and convulsions preceded death. Doses higher than the LD_50_ (24 and 48 μg protein/g) induced symptoms similar to those of the lower doses, although in most cases, deaths occurred in a shorter time. The denatured aqueous extract of *M. alcicornis* did not have any effect in mice.

**Table 4:** Symptoms in mice induced by intravenous administration of the aqueous extract of *M. alcicornis*

Dose (μg protein/g)	Symptoms	Death time
Control	Normal behavior	No death
1.5–3	Little change, affected but calm, progressive malaise, and burning	No death
6–12	Progressive agitation, respiratory difficulty, despair, malaise, later slowness, muscle paralysis of the hind leg, respiratory failure, convulsions and death; intestinal inflammation, uremia, hemolysis in the eyes and nails	Mainly within 10 h
24–48	Respiratory difficulty, hypoactivity, paralysis of the hind leg, intestinal inflammation, uremia, redness of eyes and nails, respiratory failure, convulsions, and death	Mainly within 3 h

### Histopathological analysis

**Fig. 5:**
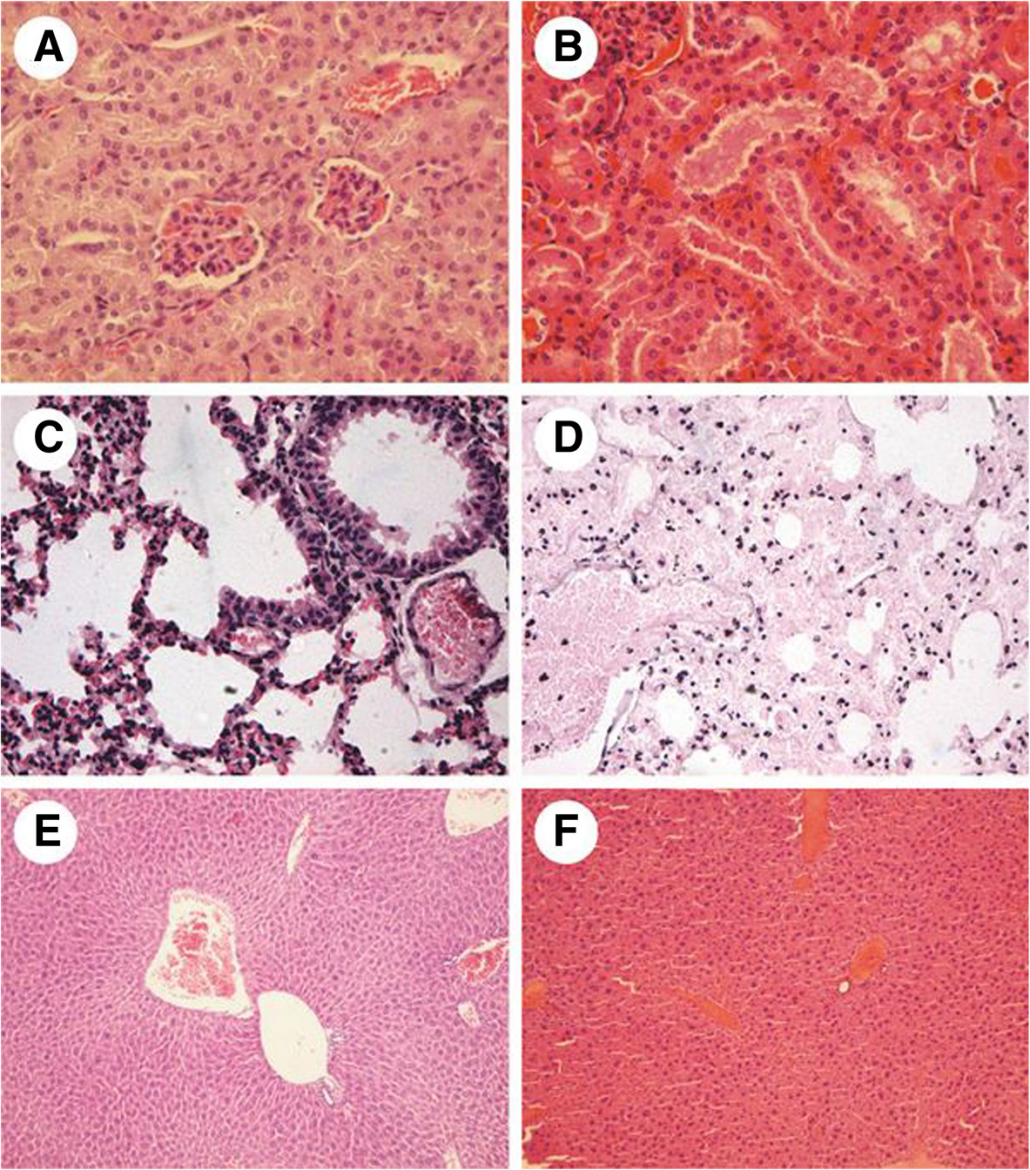
Light micrographs of tissues sections after the administration the *M. alcicornis* aqueous extract by intravenous route. **a** Control kidney section compared with a (**b**) kidney section of mice previously administrated with the extract, note severe peritubular capillary congestion. **c** Control lung section compared with a (**d**) lung section of mice previously administrated with the extract, note alveolar membrane thickening and hemorrhagic zones. **e** Control liver section compared with a (**f**) liver section of mice previously administrated with the extract, note acute vascular congestion and areas with fibrinoid material. **a**, **b**, **c** and **d**: 400× magnification. **e** and **f**: 100× magnification. Hematoxylin-eosin stain

LM images showed histopathological changes in kidney, lung and liver tissues but not in heart, brain or skeletal muscle. In kidney sections, glomerular capillaries appeared congested with proteinaceous material within the tubules. In some cases, acute tubular necrosis (Fig. 5a and b) was observed. Lung sections showed severe alveolar epithelial cells degeneration and desquamation, damage to capillaries, and marked infiltration of erythrocytes and proteinaceous material within alveoli (Fig. 5c and d). Liver sections showed varying degrees of pathological lesions, with acute vascular congestion and areas with fibrinoid material mixed with neutrophils and cell apoptosis, which indicated the development of moderate inflammatory reactions and activation of the complement system (Fig. 5e and f). Some bloods samples were taken from mice 4 h after the administration of the extract for quantification of alanine aminotransferase and aspartate aminotransferase. Unfortunately, these experiments failed since all blood samples were completely hemolysed. Injection of the denatured extract did not cause any histopathological damage in the tissues examined.

### Lethality assay in *Artemia salina*

The aqueous extract of *M. alcicornis* was toxic to *A. salina* with a LD_50_ = 70.71 μg protein/mL. It is important to point out that crude extracts are considered toxic when their LD_50_ is lower than 1000 μg/mL [20], and the denatured aqueous extract had a LD_50_ close to this value (LD_50_ = 960.28 μg protein/mL).

### Analysis by electron microscopy techniques

**Fig. 6:**
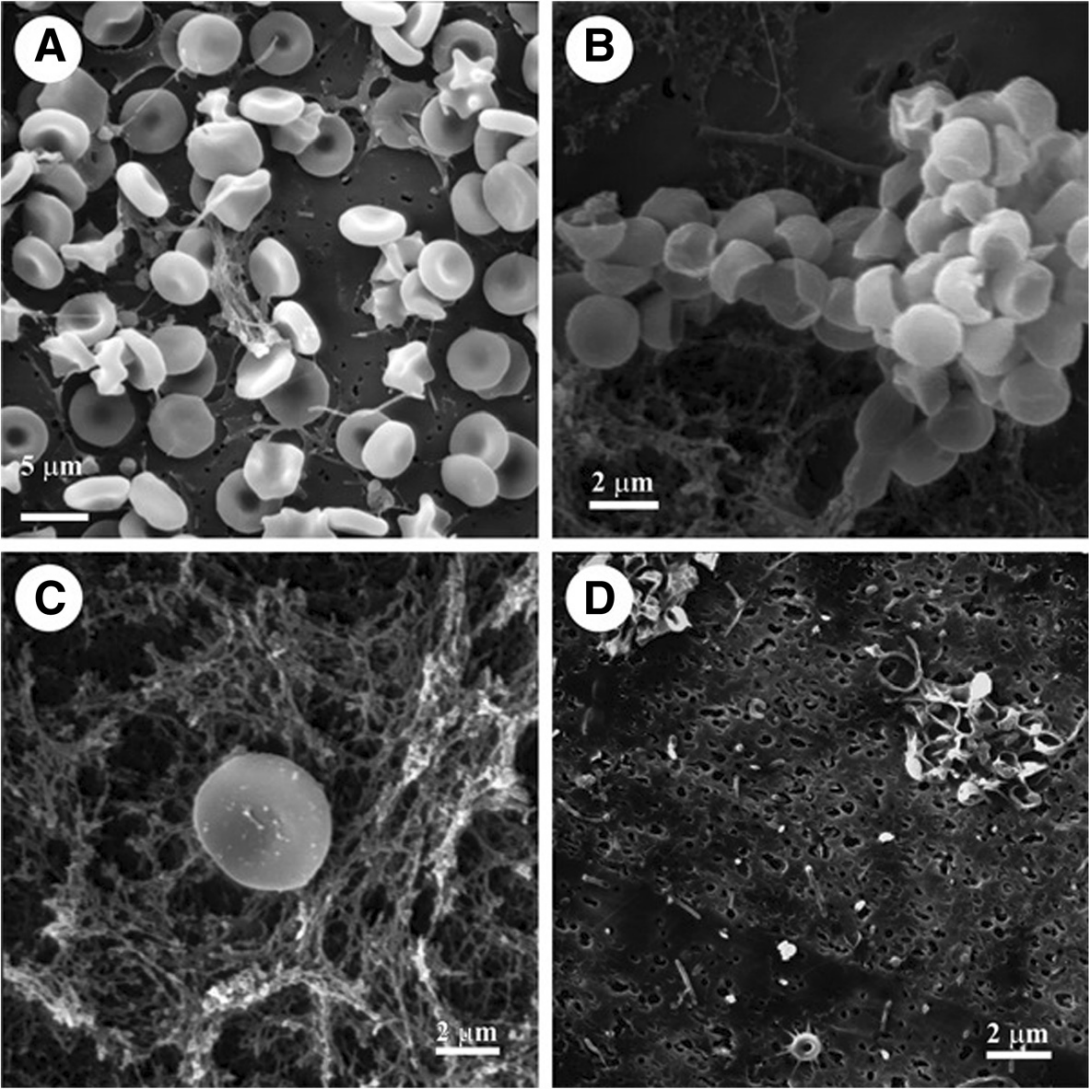
**a** Control erythrocytes preincubated with Alsever’s solution. **b** and **c** Erythrocytes preincubated with an HU_50_ of the *M. alcicornis* aqueous extract, note the amorphous material from the broken-down membranes in the background. **d** Remains of the erythrocytes membranes completely destroyed after incubation in the presence of 10-fold the HU_50_ of the *M. alcicornis* aqueous extract

**Fig. 7:**
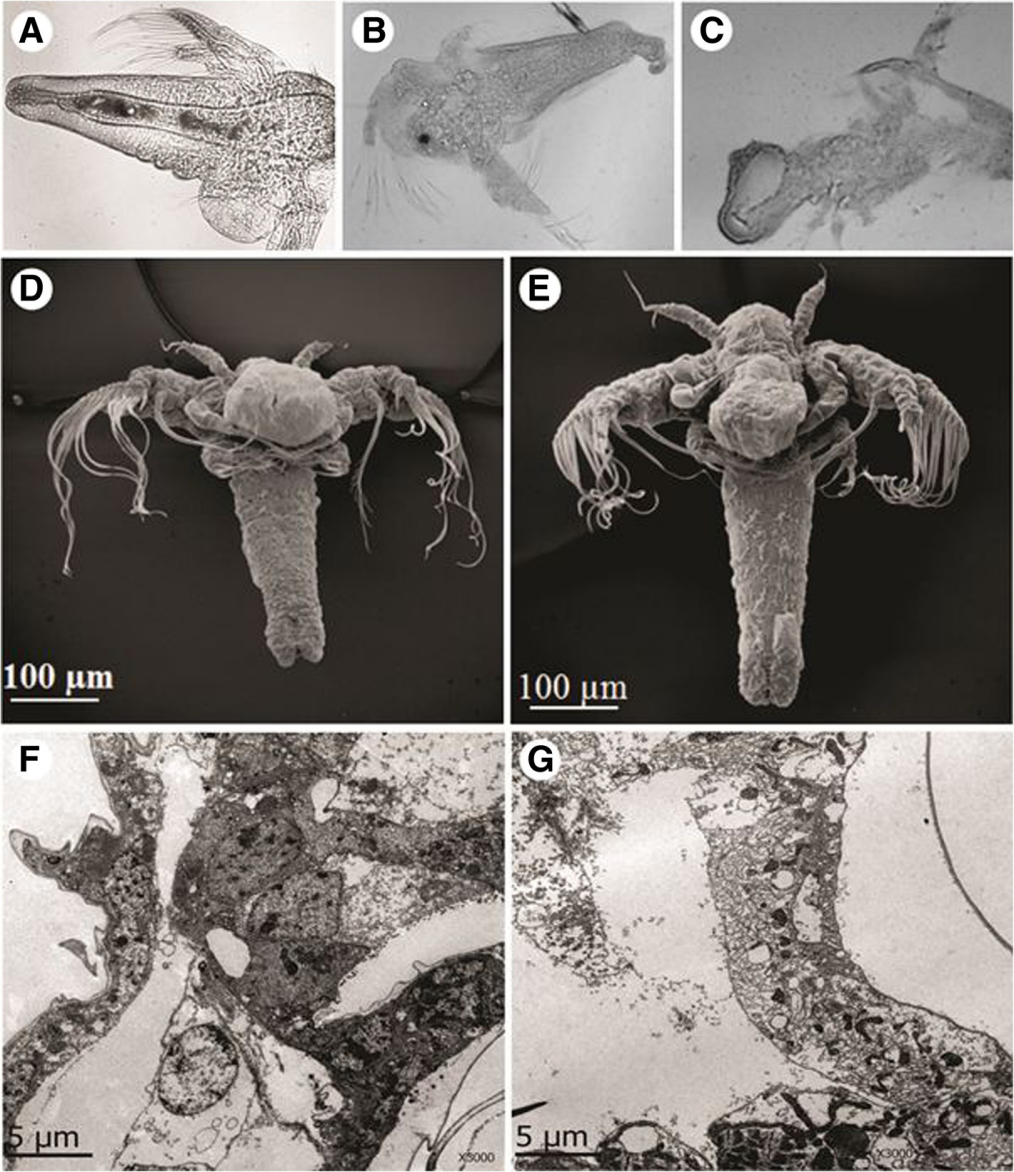
**a** *Artemia salina* brine shrimp used as control, after 24-h incubation in seawater. **b** and **c** Brine shrimps after 24 h of incubation with 10-fold the LD_50_ of the *M. alcicornis* aqueous extract; membranes are partially disintegrated. Light microscope, 400× magnification. **d** Control and (**e**) treated brine shrimp by SEM. Ultrathin longitudinal sections of (**f**) control and (**g**) treated brine shrimps by TEM

Analysis by SEM revealed that the *M. alcicornis* aqueous extract broke the erythrocyte membranes, which provoked release of intracellular material from the cells (Fig. 6). However, control erythrocytes remained viable and intact following 24 h of incubation. Microscopic analysis by optic microscopy showed that control brine shrimps had a normal cuticle and their visceral mass appeared well defined (Fig. 7a). In contrast, the visceral mass of the main body cavity of the *A. salina* nauplii was disturbed and their cuticle was damaged in the presence of the aqueous extract (Fig. 7b and c). Microscopic analysis by SEM (Fig. 7d and e) and ultrathin longitudinal sections, analyzed by TEM (Fig. 7f and g) showed cell disorganization in treated nauplii exposed to the *M. alcicornis* aqueous extract.

## Discussion

*Millepora* species are recognized for the painful stings they can inflict on human skin. However, up to now, the mechanism of the envenomation caused by these species has not been completely defined. Hemolysis is a well-known effect induced by cnidarian cytolysins, including those produced by hydrocorals. These cytolysins have also the capacity to affect other cell types and provoke damage such as inflammation and dermonecrosis [22]. In the present study, the hemolysis caused by the aqueous extract of the “fire coral” *M. alcicornis* was tested on rat, human, mouse, chicken, guinea pig, and rabbit erythrocytes. Our results showed that rodent erythrocytes were the most sensitive to the hydrocoral aqueous extract, whereas chicken erythrocytes were significantly less susceptible. It is very likely that differences observed in the erythrocytes susceptibility to lysis depend on their lipid membrane composition. In this regard, studies on the mechanism of insertion of cnidarian pore-forming cytolysins have shown that actinoporins (20 kDa proteins) irreversibly bind with high affinity to membranes containing sphingomyelin, whereas other types of cytolytic peptides (5–8 kDa) show a preference for phosphatidylcholine-containing membranes [23]. A variety of novel pore-forming cytolysins have been isolated from cnidarians and their activity strongly depends on the lipid membrane composition [22, 24, 25].

Concerning cytolysins with PLA_2_ activity, kinetic studies have revealed that these enzymes can display different affinities for membranes depending on the phospholipid composition [26]. In a previous study, regarding characterization of the hemolytic activity of the aqueous extract of *M. complanata* collected in the Mexican Caribbean, we found a similar behavior on the erythrocytes susceptibility to lysis [27], which suggests that cytolysins produced by both Caribbean hydrocorals have a high similarity in the membrane-binding domains. These results are in contrast with those found by Shiomi et al. [9], who tested the hemolytic activity of *M. dichotoma* and *M. platyphylla* venoms on cow, horse, sheep, rabbit, guinea pig, mouse, and chicken erythrocytes. These researchers observed that horse erythrocytes were highly sensitive to the *M. dichotoma* venom, while they were much less sensitive to the *M. plathyphylla* venom. Conversely, chicken erythrocytes were more sensitive to the *M. plathyphylla* venom than to that of *M. dichotoma*.

In this study we also found that the hemolytic activity induced by the extract of *M. alcicornis* was progressively reduced when it was preincubated at temperatures above 45 °C and was completely lost after preincubation at temperatures over 70 °C. These results indicate that the hemolysins contained in this extract are mainly thermo-labile proteins. These hemolysins are relatively stable in a range of incubation temperatures from 30 to 43 °C. At temperatures lower than 30 °C, the hemolytic activity of the aqueous extract was reduced, which indicates that warm temperatures stimulate the hemolytic reaction. Furthermore, the *M. alcicornis* hemolysins also showed high stability to pH variation, since their activity was preserved in the range from 2 to 12. These results differ from those observed in a study performed with an extract prepared from *M. alcicornis* collected on the beaches in Miami [15]. In that study, it was found that the extract preserved its activity in a pH range of 5.5 to 8.7, but lost its activity at a pH of 4.8. These differences confirm that toxin synthesis highly depends on the environmental conditions.

It has also been reported that hemolytic activity of cnidarian venoms exhibits a differentiated dependence on divalent cations, and particularly, cnidarian phospholipases A_2_ have a Ca^2+^ binding loop, which is highly conserved [28]. Regarding cnidarian pore-forming toxins, it has been demonstrated that Ca^2+^ is necessary for the dimerization of the toxin within the membrane [29], while other cnidarian pore-forming toxins can be inhibited by Zn^2+^ [30]. In this study, we found that the hemolytic activity of the aqueous extract of *M. alcicornis* was increased in the presence of ≥ 0.01 mM (Ca^2+^, Mg^2+^, or Ba^2+^) and was reduced in the presence of Cu^2+^ (≥0.1 mM), Zn^2+^ (≥6 mM), and EDTA (≥0.43 mM). These results indicate that divalent cations modify the hemolytic activity induced by the aqueous extract of this “fire coral”. In the case of the inhibition by EDTA, this effect might be attributed to the chelation of Ca^2+^ and Mg^2+^. These findings differ from those obtained in our previous study, in which we examined the effect of Ca^2+^ and Mg^2+^ on the hemolytic activity induced by the aqueous extract of *M. complanata* [27], which suggest structural differences between the Ca^2+^ binding loop of the cytolysins produced by these *Millepora* species. It is worth mentioning that inhibition of the cytolytic activity by Cu^2+^, Zn^2+^, and EDTA could be useful for the development of adequate treatments for stings caused by the “fire corals”. Previous studies have shown that zinc gluconate inhibits potassium efflux from erythrocytes exposed to venom or purified hemolysin from *Chironex fleckeri* and prolongs survival time in mice injected with the venom. Therefore, administration of this ion has been considered useful in the treatment of victims of *C. fleckeri* stings [30].

Up to now, the hemolytic mechanisms of *Millepora* venoms have not been completely characterized, but previous studies suggest that PLA_2_ proteins significantly contribute to this effect. In this context, it has been reported that *Millepora* species possesses higher PLA_2_ activity levels than other cnidarians [31]. In a previous study, we found that the hemolytic activity of the aqueous extract of *M. complanata* significantly decreased after incubation with the PLA_2_ inhibitor *p*-bromophenacyl bromide, and we detected a hemolysin with a molecular weight similar to that of milleporin-1, a PLA_2_ isolated in the venom of *M. platyphylla* [32]. We also found that the aqueous extract of *M. complanata* has PLA_2_ activity, but not protease activity [33] Accordingly, the results obtained in this study showed that the hemolysis produced by the aqueous extract of *M. alcicornis* was significantly inhibited, but not completely blocked by *p*-bromophenacyl and it does not contain proteases. These findings indicate that toxins with PLA_2_ activity significantly contribute to the overall hemolysis induced by this hydrocoral extract. However, it is very likely that this extract also contains pore-forming toxins, since the hemolytic activity of the extract was not completely suppressed with the PLA_2_ inhibitor.

Electrophoretic analysis revealed that the *M. alcicornis* extract contains proteins with a broad range of molecular weights. When the electrophoresis gel, run under non-reduction conditions, was incubated on blood agar gel, two bands displayed hemolytic activity: an approximately 28–30 kDa-band and an approximately 200 kDa-band. The last one was not observed when electrophoresis was run under reducing conditions, which suggests that these high molecular weight proteins could be polymers. The PLA_2_ zymograms showed that the hemolytic 28–30 kDa-band also induces PLA_2_ activity, whereas the 200-kDa band region did not have enzymatic activity, suggesting the presence of pore-forming polymeric cytolysins. This type of toxin has been described in venoms of cnidarians of the class Hydrozoa. For example, physalitoxin is a potent hemolysin of 240 kDa detected in the *Physalia physalis* venom [34].

In another set of experiments, the systemic toxicity of the extract was assessed in mice by intravenous administration. Previous studies showed that the venom of *M. alcicornis* caused extensive hemolysis and death in mice after several hours [15]. In the case of this study, similar symptoms were observed, which consisted in generalized hemolysis, forced respiration, convulsions, and death; depending on the dosage. However, the lethality of the extract prepared from *M. alcicornis* specimens recently collected in the Mexican Caribbean was lower (LD_50_ = 17 μg protein/g) than that produced by the specimens previously studied (LD_50_ = 0.55 and 1.136 μg protein/g). These differences might be attributed to environmental changes, since the specimens were collected in different zones and times, which results in variations in toxins production. Interestingly, important differences were found between the toxicity induced by the *M. alcicornis* aqueous extract and that provoked by the *M. complanata* extract [33], both species collected in the Mexican Caribbean. In our previous study, we observed that the aqueous extract of *M. complanata* induced two types of death in mice after its intravenous administration: at doses higher than the LD_50_ (LD_50_ = 4.62 μg protein/g) the extract produced violent convulsions and death within 1 min; however, lower doses induced a slow death similar to that provoked by the *M. alcicornis* extract. After denaturation by heating, the *M. complanata* extract completely lost its capacity to induce the slow death, but conserved its immediate lethal effect [33]. In contrast, the denatured extract of *M. alcicornis* did not induce any response in mice, which suggests that the compounds responsible for the systemic toxicity induced by the *M. alcicornis* extract are heat-labile proteins, whereas the *M. complanata* extract also contains thermo-stable toxins that are capable of inducing a rapid death in mice.

Histophatological analysis of tissues dissected from mice administrated with the aqueous extract of *M. alcicornis* revealed important damage to the lung, liver and kidneys. Similar effects have been observed in mice after intravenous administration of a tentacle extract from the jellyfish *Cyanea capillata*. At high doses, mice deaths occurred within 2 h probably due to acute heart-or-nervous-related toxicities. At lower doses, deaths occurred between 2 and 48 h with acute liver and renal failure. That toxin-induced multiple organ dysfunction was defined as the “delayed jellyfish envenomation syndrome”, whose etiology has not been completely elucidated, but it has been related to the synergy of various toxic mechanisms caused by a variety of toxins contained in the jellyfish venom [35]. In the present research, it was found that the aqueous extract of *M. alcicornis* induced a delayed envenomation syndrome similar to those elicited by the venom of *C. capillata* and the aqueous extract of *M. complanata* [33]. It is likely that these toxic effects are caused mainly by cytolysins that disrupt several cell types, including those of epithelial tissues. It has been observed that epithelial cells of nephrons can be markedly affected by cytolysins [36], since kidneys represent the main route of elimination of toxins. Therefore, the hemoglobinuria caused by the *M. alcicornis* extract in the mice could be a consequence of both renal damage and hemolysis. Moreover, we have previously found that the aqueous extracts of *M. alcicornis* and *M. complanata* provoke vasoconstriction on isolated rat aortic rings [14]. This vasoconstrictor effect could also trigger multiple organ injuries as a consequence of compensatory responses due to ischemia or hypoxia. These systemic toxic effects observed in mice could explain the fact that human contact with these hydrocorals can result in renal disease [5].

A cytotoxicity assay using *A. salina* was carried out to further analyze the toxicity of the aqueous extract of *M. alcicornis*. This extract was lethal to *A. salina* nauplii (LD_50_ = 70.71 μg protein/mL), which is not surprising since small crustaceans are part of the diet of *Millepora* hydrocorals [1]. The denatured extract was significantly less lethal (LD_50_ = 960.28 μg protein/mL), indicating that the cytotoxic compounds are mainly thermolabile proteins. Microscopic analysis of brine shrimps incubated in the presence of the extract showed tissue dissociation. This effect is usually attributed to pore-forming cytolysins, which form pores in the membranes of these crustaceans, lysing the cells and disintegrating tissues in order to facilitate phagocytosis and the subsequent digestion of the prey [37].

## Conclusions

The results of the present study demonstrate that the aqueous extract of *M. alcicornis* contains hemolytic proteins that are thermolabile, stable to pH changes, and whose activity is calcium-dependent. This study also evidenced the presence of two types of cytolysins in this extract: some of them consist of proteins of approximately 28–30 kDa with PLA_2_ activity, while the others are larger proteins of approximately 200 kDa, which do not possess PLA_2_ activity and possibly act by a pore-forming mechanism. In addition, it was found that this hydrocoral extract induces damage in lung, kidneys and liver tissues, which results in a slow death of mice. Further research is in progress to purify and characterize the individual hemolytic compounds of the aqueous extract of *M. alcicornis*.

### Ethics committee approval

All experiments were performed in accordance with the Mexican Official Standard NOM-062-ZOO-1999 for the production, care and use of laboratory animals. Care and use of animals was approved by the Bioethics Committee of the School of Medicine, UAQ.
